# Toxicity Assessment of 6-Deoxytetrodotoxin by Mouse Bioassay and Its Distribution in Pufferfish

**DOI:** 10.3390/md24070250

**Published:** 2026-07-18

**Authors:** Yuta Kudo, Mari Yotsu-Yamashita

**Affiliations:** 1 Frontier Research Institute for Interdisciplinary Sciences, Tohoku University, 6-3 Aramaki-Aza-Aoba, Aoba-ku, Sendai 980-8578, Japan; 2 Graduate School of Agricultural Science, Tohoku University, 468-1 Aramaki-Aza-Aoba, Aoba-ku, Sendai 980-8572, Japan

**Keywords:** tetrodotoxin, 6-deoxytetrodotoxin, pufferfish, toxicity, mouse bioassay, LC-MS, food poisoning

## Abstract

Tetrodotoxin (TTX, **1**) is a potent neurotoxin that occurs in a wide range of marine and terrestrial organisms. 6-DeoxyTTX (**2**) was isolated from pufferfish as a low-abundance TTX analogue. Although the toxicities of major TTX analogues have been investigated, the acute toxicity and biological distribution of 6-deoxyTTX remain poorly understood because of limited availability of purified or synthetic 6-deoxyTTX. In this study, acute toxicity of 6-deoxyTTX was evaluated based on mouse bioassay. The toxicity of 6-deoxyTTX was estimated to be 1 MU = 570 ng, with an LD_99_ value of 28 μg/kg. Based on comparison with the LD_99_ value of TTX (10 μg/kg), the relative potency of 6-deoxyTTX was estimated to be 0.36, indicating higher toxicity than other deoxy analogues. In addition, high-resolution LC–MS analysis revealed the occurrence of 6-deoxyTTX in toxic pufferfish species in the genera *Takifugu*, *Arothron*, and *Lagocephalus*. Although 6-deoxyTTX was generally present at much lower concentrations than TTX, 6-deoxyTTX should be considered a toxicologically relevant analogue because of its relatively high acute toxicity. These results demonstrate that 6-deoxyTTX is a low-abundance TTX analogue with relatively high toxicity, providing new information relevant to food safety assessment and the distribution of TTX analogues in pufferfish.

## 1. Introduction

**Figure 1:**
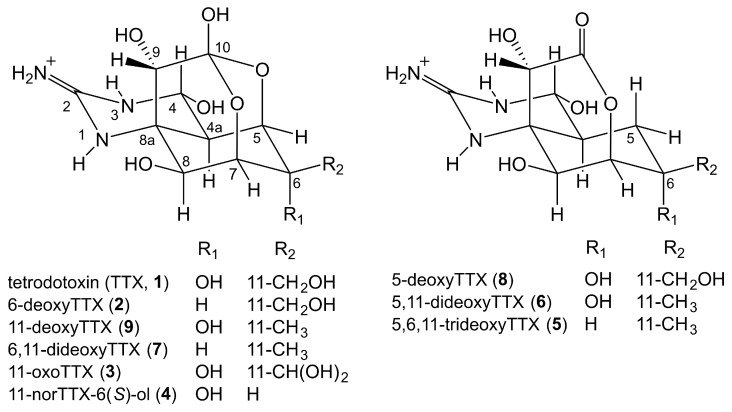
Chemical structures of tetrodotoxin (TTX, **1**) and its natural analogues (**2**–**9**) identified in marine organisms, including pufferfish.

Tetrodotoxin (TTX, **1**, Figure 1) is one of the best-known natural toxins and acts as a highly potent and selective blocker of voltage-gated sodium channels (Na_v_s). Structurally, TTX possesses a unique and highly complex molecular architecture comprising a 2,4-dioxaadamantane skeleton and a cyclic guanidinium moiety. Since its first isolation from pufferfish, TTX has subsequently been identified in a wide range of marine and terrestrial organisms, including crabs, gastropods, blue-ringed octopuses, sea slugs, flatworms, newts, toads, frogs and oribatid mites, demonstrating a remarkably broad taxonomic and geographic distribution [1,2,3,4,5,6,7,8,9]. The absence of TTX in cultured pufferfish and captive-reared newts suggested that these animals do not biosynthesize the toxin themselves but instead acquire and accumulate it from external sources [10,11,12,13,14]. Consistent with this hypothesis, transfer of TTX has been proposed in several food webs, including predator–prey relationships [6,15,16,17,18]. Among the organisms implicated in TTX transfer, toxic flatworms have emerged as important candidates because of their high toxin content and potential role as prey for TTX-bearing animals, including pufferfish [17,19,20,21,22,23]. Yasumoto et al. and Noguchi et al. first reported bacterial origin of TTX [24,25]. After that, numerous studies have suggested a bacterial origin of TTX; however, no microorganism capable of stable, high-level TTX production has been established [6,26,27]. Many aspects of its ecological transfer through food webs and accumulation pathways are also still poorly understood.

TTX causes fatal seafood poisoning following the consumption of toxic marine organisms, particularly pufferfish and gastropods, and numerous intoxication cases have been reported, mainly in East Asia, including Japan, China, Taiwan, Singapore, and Bangladesh [28,29]. More recently, the Indo-Pacific pufferfish *Lagocephalus sceleratus* has invaded the Mediterranean Sea, and a case of poisoning following its consumption was reported in Lebanon in 2022 [30]. Suspected TTX poisoning caused by pufferfish has also been documented in Arab countries [31]. In addition, pufferfish poisoning attributed to TTX has been reported in countries bordering the Indian Ocean and the Red Sea, indicating that TTX intoxication is not limited to East Asia and is of growing concern in other regions [32]. Notably, TTX has been detected in bivalves including scallops and oysters in Japan, China, New Zealand, and Europe, attracting increasing attention from a food safety perspective [33,34,35,36,37,38,39,40,41,42,43,44]. In a risk assessment conducted by the European Food Safety Authority (EFSA), a concentration below 44 μg TTX equivalents/kg of seafood was considered unlikely to cause adverse effects in humans, highlighting the importance of accurate toxicity data for TTX analogues in food safety evaluations [45].

In pufferfish and other marine organisms, both the toxic organs and TTX contents vary among species and geographic populations, and substantial individual variation is also observed, consistent with the exogenous origin of the toxin [6,46,47,48]. More complex toxification patterns have recently been reported in natural hybrid pufferfish [49,50,51,52]. Since pufferfish poisoning frequently results from the consumption of non-commercial or non-edible species, toxicological information on a broad range of pufferfish species is required for accurate risk assessment. Therefore, detailed characterization of TTX analogues, including their toxicity, occurrence, and tissue distribution in diverse pufferfish species, is of considerable importance.

In Japan, commercially important edible pufferfish species mainly belong to the genus *Takifugu*. The toxification profiles of these species have been extensively investigated [14,53,54], and edible tissues have been identified on the basis of their toxin distribution. In general, the ovary and liver are highly toxic tissues, while the skin and intestine are also recognized as major toxin-bearing organs, with tissue-specific toxicity varying among species [6,46]. Species that do not accumulate toxins in their muscle tissues are commonly consumed, whereas some pufferfish species are known to accumulate substantial amounts of TTX in their muscles. In addition, some pufferfish species have been reported to contain not only TTXs but also the representative toxin of paralytic shellfish toxins, saxitoxin (STX), highlighting the chemical complexity of pufferfish toxins and the need for comprehensive toxin profiling [55,56,57,58].

Current pufferfish toxin analyses have focused primarily on TTX itself, whereas detailed compositional analyses of the various TTX analogues remain limited because authentic standards are unavailable for most compounds. However, accurate risk assessment of pufferfish poisoning requires not only quantification of TTX but also characterization of the distribution and composition of TTX analogues with different toxic potencies. EFSA has proposed relative potency factors for several TTX analogues for food safety risk assessment. Although most analogues were estimated to be less toxic than TTX, EFSA noted that these estimates are associated with considerable uncertainty because of the limited toxicological data available for many analogues [45]. Various analytical methods, including post-column liquid chromatography–fluorescence detection (LC-FLD) [59,60], competitive ELISA [61,62,63], and LC–MS [64,65,66], have been developed for TTX analysis. Among them, high-resolution (HR)-HILIC–LC–MS is a powerful approach for the simultaneous identification and compositional analysis of multiple TTX analogues [67]. Using LC–MS, Yotsu-Yamashita and co-workers reported the composition and tissue distribution of TTX analogues in several pufferfish species, revealing diversity in analogue profiles among species and tissues [65,67,68].

TTX exerts potent neurotoxicity by blocking TTX-sensitive subtypes of Na_v_s, thereby preventing the initiation and propagation of action potentials in excitable cells. The interaction between TTX and Na_v_ channels has been studied for decades, and numerous studies have examined their structure–activity relationships and binding modes. Various TTX analogues have been identified from TTX-bearing organisms, and studies of their biological activities have provided important insights into both the structure–activity relationships underlying Na_v_ inhibition and the risk assessment of TTX-related food poisoning (see also Discussion) [69,70,71,72,73]. Cryo-electron microscopy (cryo-EM) structures of TTX bound to Na_v_ were reported [74,75], confirming the importance of its guanidinium and hydroxyl groups in channel binding. These structural findings are in good agreement with the structure–activity relationships that had been established through earlier pharmacological and biochemical studies.

Although the profiles of TTX analogues differ substantially between marine organisms and toxic terrestrial newts [76,77], TTX is generally considered the principal toxic component in both groups because of its high potency and abundance. Among the major analogues, 4-*epi*TTX and 4,9-anhydroTTX exist in equilibrium with TTX but exhibit lower Na_v_ inhibitory activities, as demonstrated by receptor-binding assays using rat brain membrane preparations and electrophysiological analyses [70,78]. In contrast, 11-oxoTTX (**3**), which has been identified in certain pufferfish, gastropods, and terrestrial amphibians [79,80,81], exhibits potency comparable to that of TTX in several assays [70,82]. Marine TTX analogue, 11-norTTX-6(*S*)-ol (**4**), also exhibits appreciable toxicity [70,78]. More recently, the toxicities of these analogues were evaluated by mouse bioassays using highly purified samples [78]. Several deoxyTTX analogues lacking hydroxyl groups at the C-5, C-6, and/or C-11 positions have been reported from marine organisms, namely 5,6,11-trideoxyTTX (**5**), 5,11-dideoxyTTX (**6**), 6,11-dideoxyTTX (**7**), 5-deoxyTTX (**8**), 6-deoxyTTX (**2**), and 11-deoxyTTX (**9**) (Figure 1) [67,83,84,85,86,87,88]. In general, 5-deoxy-type analogues exhibit markedly reduced activity because they lack the C-10 orthoester moiety that is characteristic of TTX and highly toxic analogues. Likewise, 11-deoxyTTX exhibits lower toxicity and weaker Na_v_ inhibitory activity than TTX, although it still retains measurable activity [70,86,87]. The representative deoxy analogue 5,6,11-trideoxyTTX (**5**) has been detected in a wide range of marine organisms and has been reported to function as a chemical attractant for pufferfish, suggesting biological roles of TTX analogues beyond Na_v_ inhibition [89,90]. In addition, several stereoisomeric analogues of TTX, including 6-*epi*TTX (**10**), 6-*epi*-11-deoxyTTX (**11**), 8-*epi*TTX from newt, and 9-*epi*TTX from pufferfish, have been isolated and their biological activities have been investigated [73,77,87,91].

Among the known TTX analogues, 6-deoxyTTX (**2**) was originally isolated in a small amount (40 μg) from 250 g of ovaries of the Japanese pufferfish *Takifugu pardalis* through multiple chromatographic purification steps including HILIC [86]. Because only trace amounts have been obtained from natural sources and neither commercial nor synthetic standards are available, information regarding the occurrence and toxicity of this analogue remains limited. 6-DeoxyTTX (**2**) has also been detected in the toxic gastropod *Nassarius glans* and in the salivary glands of blue-ringed octopuses (*Hapalochlaena* spp.) [86]. To date, 6-deoxyTTX has been reported to exhibit approximately one-third the activity of TTX in the Neuro-2a assay [86], which evaluates Na_v_ inhibition based on protection against cytotoxicity induced by ouabain, a Na^+^/K^+^-ATPase inhibitor, and veratridine, a persistent activator of Na_v_s [92]. This finding suggests that 6-deoxyTTX (**2**) has substantial Na_v_-inhibitory activity. However, its toxicity to mice has not been evaluated, and its toxicological properties remain largely unknown. Furthermore, the occurrence and abundance of 6-deoxyTTX in pufferfish, the representative causative organisms of TTX poisoning, remain unknown. To assess the contribution of this analogue to pufferfish toxicity, it is necessary not only to evaluate its toxicity but also to determine its abundance and tissue distribution across diverse pufferfish species.

In this study, we performed the first direct evaluation of the acute toxicity of 6-deoxyTTX (**2**) using the mouse bioassay, an official standard method for assessing pufferfish toxicity in Japan [93], despite the absence of commercially available or synthetic standards. In addition, TTX and 6-deoxyTTX (**2**) were analyzed by HR-HILIC–LC–MS in the skin, liver, and gonads of 10 pufferfish species belonging to the genera *Takifugu*, *Arothron*, and *Lagocephalus*, and a TTX-bearing marine flatworm. Based on these results, we evaluated the potential contribution of 6-deoxyTTX (**2**) to the overall toxicity associated with pufferfish poisoning. 

## 2. Results

### 2.1. Mouse Bioassay of 6-deoxyTTX (***2***)

Because of the limited amount of 6-deoxyTTX (**2**) available, the purified sample obtained after HILIC chromatography had a purity of 87.9% (Figure S1). TTX was below the limit of detection (LOD; see Materials and Methods) in this sample. The major impurities were 4-*epi*TTX (3.8%) and 4,4a-anhydroTTX (8.3%), both of which have lower reported Na_v_ inhibitory activities than TTX [78,82], suggesting that their contribution to the observed toxicity was limited.

The results of the mouse bioassay are summarized in Table 1. Owing to the limited quantity of purified 6-deoxyTTX (**2**), the assay was conducted using five male mice (15–16 g body weight). The observed symptoms, including reduced activity, ataxia, convulsions, abnormal motor activity, running around, jumping, and gasping respiration, were consistent with previously reported TTX intoxication symptoms in experimental animals [45,93]. Intraperitoneal (i.p.) administration of 1500 ng and 1000 ng of 6-deoxyTTX (**2**) caused typical symptoms of TTX intoxication and resulted in death after 4 min 24 s and 7 min 3 s, respectively. At a dose of 750 ng, one mouse died after 14 min, whereas the second mouse exhibited severe intoxication symptoms but gradually recovered after 30 min and survived for 24 h (end point). Administration of 500 ng also induced intoxication symptoms, although the mouse recovered after 30 min and survived for 24 h. No delayed death was observed in mice that recovered after 30 min.

One mouse unit (MU) is defined as the amount of toxin required to kill a 20-g mouse within 30 min and corresponds to 220 ng of TTX [93,94]. In this study, the MU value for 6-deoxyTTX (**2**) was derived using the official tetrodotoxin toxicity conversion table specified in the Japanese standard method for pufferfish toxin analysis [93], taking into account the body weight and time to death of each mouse. The MU of 6-deoxyTTX (**2**) was estimated to be 570 ng, approximately 2.5-fold greater than that of TTX. The LD_99_ value, calculated as [minimum lethal dose (μg) × 50/MU], was estimated to be 28 μg/kg based on the average of lethal cases. This value was approximately three-fold higher than the reported LD_99_ values of TTX (10–12 μg/kg) [45,78,93,94,95].

### 2.2. Concentrations of TTX and 6-deoxyTTX (***2***) in Pufferfish

**Figure 2:**
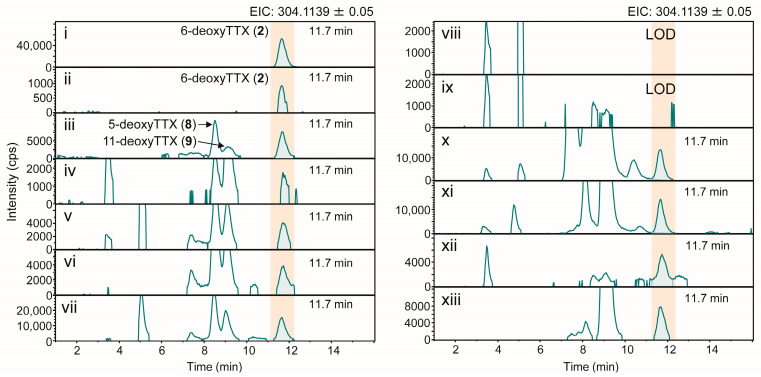
Representative HR-HILIC–LC–MS extracted ion chromatograms of 6-deoxyTTX (**2**) standards and the extracts from pufferfish tissues and flatworm. (**i**) 6-deoxyTTX standard (250 pg); (**ii**) 6-deoxyTTX standard (5 pg); (**iii**) *Takifugu pardalis* liver; (**iv**) *T. snyderi* skin; (**v**) *T. niphobles* ovary; (**vi**) *T. exascurus* ovary; (**vii**) *T. flavipterus* ovary; (**viii**) *T. rubripes* skin; (**ix**) *T. vermicularis* skin; (**x**) *Arothron hispidus* skin; (**xi**) *A. manilensis* skin; (**xii**) *Lagocephalus sceleratus* skin; and (**xiii**) planocerid flatworm. The area under the 6-deoxyTTX peak is filled, and the corresponding retention-time region is highlighted.

The results of toxin analyses are summarized in Table 2. Both TTX (**1**) and 6-deoxyTTX (**2**) were detected in pufferfish belonging to all three genera examined in this study (*Takifugu*, *Arothron*, and *Lagocephalus*). TTX was detected in all 20 individuals representing the ten pufferfish species examined. In contrast, 6-deoxyTTX (**2**) was detected in eight species. Representative HR-HILIC–LC–MS chromatograms of each pufferfish species are shown in Figure 2. The measured exact masses agreed with the theoretical value within 4 mDa.

In wild *T. rubripes*, one of the major edible pufferfish species in Japan, skin, muscle, and liver tissues from two individuals were analyzed. TTX was detected only at trace levels, with 0.093 μg/g in the skin of one individual and 0.13 μg/g in the liver of the other individual; all other skin, muscle, and liver samples were below the LOQ, although TTX accumulation in the liver of this species has been reported in many previous studies [6,46,96]. Furthermore, 6-deoxyTTX (**2**) was not detected in any of the *T. rubripes* tissues examined. In three *T. vermicularis* individuals, TTX was detected in the skin at concentrations of 0.71–1.8 μg/g, whereas 6-deoxyTTX (**2**) was not detected in any of the six skin and liver samples examined. 

Excluding *T. rubripes* and *T. vermicularis*, 6-deoxyTTX (**2**) was detected in 37 of the 44 tissue samples from the remaining eight pufferfish species, indicating its widespread distribution among toxic pufferfish. However, the concentrations of 6-deoxyTTX (**2**) were substantially lower than those of TTX. Seventeen samples contained more than 100 ng/g of 6-deoxyTTX (**2**), and the highest concentration observed was 1802 ng/g in the skin of *A*. *manilensis*, where the corresponding TTX concentration was 78 μg/g. The proportion of 6-deoxyTTX (**2**) relative to TTX was generally low, ranging from 0.23% to 13.4% (w/w) with a mean value of 3.2% (median 2.7%, *n* = 33). The highest proportion (13.4%) was observed in the liver of *T*. *pardalis*, followed by 6.3% in the skin of *Lagocephalus sceleratus* and 5.7% in the ovary of *T. pardalis*. No pufferfish species or tissue showed preferential accumulation of 6-deoxyTTX (**2**). Furthermore, 6-deoxyTTX (**2**) was also detected in the toxic flatworm *Planocerid* sp. [5]. However, its abundance was similarly low, accounting for only approximately 2% of the TTX content.

## 3. Discussion

### 3.1. Acute Toxicity and Relative Potency of 6-deoxyTTX (***2***)

The toxicological evaluation and distribution analysis of TTX analogues remain important issues in tetrodotoxin research. Although 6-deoxyTTX (**2**) has been detected in several toxic organisms and was suggested to possess relatively high Na_v_ inhibitory activity [86], information regarding its acute toxicity and occurrence in biological samples has been limited.

In the present study, the potency of 6-deoxyTTX (**2**) in the mouse bioassay was estimated to correspond to 1 MU = 570 ng, with an approximate LD_99_ value of 28 μg/kg. The purified 6-deoxyTTX (**2**) used for toxicity testing contained minor amounts of 4-*epi*TTX (3.8%) and 4,4a-anhydroTTX (8.3%), whereas TTX and other known TTX analogues were not detected. The influence of these impurities on the observed toxicity is likely limited. Previous electrophysiological studies using human Na_v_1.2 reported an IC_50_ value of 180 nM for 4-*epi*TTX, compared with 11 nM for TTX [78], and its LD_99_ value in mice has been reported as 70 μg/kg [97]. In addition, 4,4a-anhydroTTX exhibited substantially lower activity in a Neuro-2a assay. Its EC_50_ value was reported to be 3300 nM, compared with 4.7 nM for TTX [82]. Considering both their low relative abundance and reduced potency, these analogues are unlikely to have substantially affected the acute toxicity observed in this study. Nevertheless, the toxicity value obtained in this study should be regarded as an approximate estimate, and further toxicity evaluation using a more highly purified 6-deoxyTTX (**2**) sample will be necessary for a more precise determination of its potency.

In 2017, the European Food Safety Authority (EFSA) reviewed the available literature on the toxicity of TTX analogues and summarized their estimated relative potencies for risk assessment [45]. In the present study, the relative potency of 6-deoxyTTX (**2**) was estimated and compared with previously reported relative potencies of TTX analogues determined by mouse bioassays and Neuro-2a assays (Table 3). TTX has been reported to have an i.p. LD_99_ value of 10–12 μg/kg [45,78,93,94,95], whereas the i.p. LD_50_ values of 11-deoxyTTX and 6,11-dideoxyTTX are 71 and 420 μg/kg, respectively [85,87]. Based on the i.p. LD_99_ value (28 μg/kg), the relative potency of 6-deoxyTTX (**2**) in the mouse bioassay was estimated to be approximately 0.36. This value was higher than those reported for several deoxy analogues, including 11-deoxyTTX (**9**), 6,11-dideoxyTTX (**7**), and 5,6,11-trideoxyTTX (**5**), with relative potencies of 0.14, 0.02, and 0.01, respectively [83,85,87]. A similar tendency was observed in the Neuro-2a assay, where 6-deoxyTTX (**2**) exhibited an EC_50_ value of 6.5 nM compared with 2.1 nM for TTX [86], corresponding to a relative potency of 0.32. This Neuro-2a assay-based relative potency of 6-deoxyTTX (**2**) was in good agreement with that estimated from the mouse bioassay. Together with previously reported data for other TTX analogues, these results suggest that 6-deoxyTTX (**2**) retains relatively high toxicity among deoxy-type TTX analogues. It should be noted, however, that because of the limited availability of purified 6-deoxyTTX (**2**), the mouse bioassay was performed with a limited number of animals. Therefore, the toxicity estimated in this study should be regarded as an approximate value rather than a definitive potency. Further evaluation with larger numbers of animals will be required for a more precise determination of its potency.

### 3.2. Structural Interpretation of the Toxicity of 6-deoxyTTX (***2***) and Related Analogues

**Figure 3:**
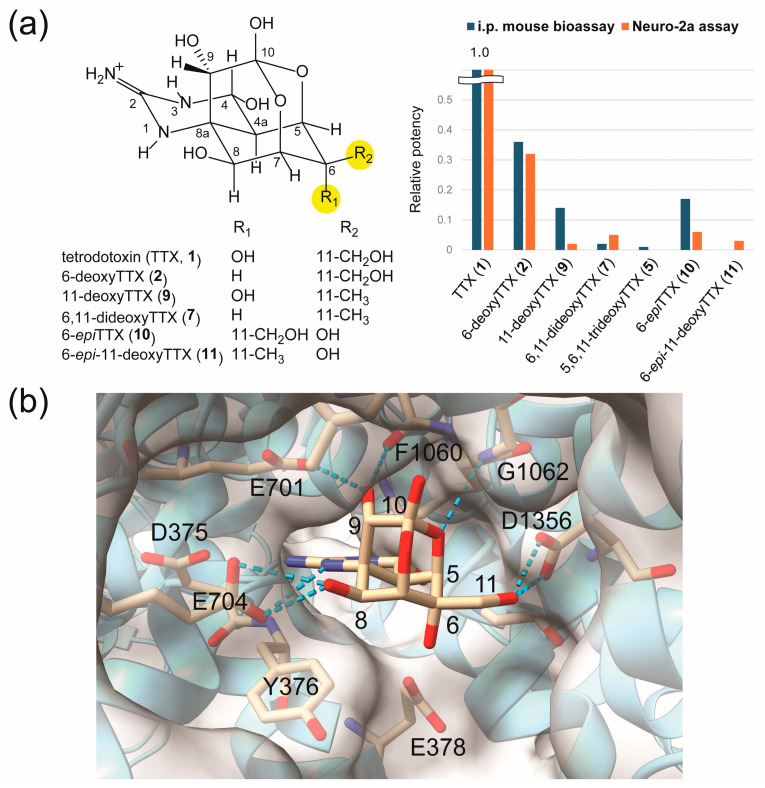
Comparison of structures and relative potencies of TTX analogues and the binding environment of TTX in Na_v_. (**a**) Structures and estimated relative potencies of TTX (**1**), 6-deoxyTTX (**2**), 11-deoxyTTX (**9**), 6,11-dideoxyTTX (**7**), 5,6,11-trideoxyTTX (**5**), 6-*epi*TTX (**10**), and 6-*epi*-11-deoxyTTX (**11**). Relative potencies were determined by i.p. mouse bioassay and/or Neuro-2a assay. Blank bars indicate values that have not been reported. (**b**) Structural environment of TTX in the outer vestibule of Na_v_PaS based on the TTX-bound Na_v_PaS structure [74]. TTX is shown as sticks, and selected surrounding residues are labeled. Cyan dashed lines indicate selected polar contacts between TTX and residues around the selectivity filter. The structure is shown to illustrate the spatial arrangement of TTX functional groups in the Na_v_ outer vestibule and does not represent a structure of 6-deoxyTTX-bound Na_v_. The Na_v_PaS structure used here is derived from an insect voltage-gated sodium channel and was used only as a structural reference to illustrate the general binding mode of TTX and its analogues in the outer vestibule of sodium channels.

Structural information provides important insights into the relative toxicities of TTX analogues. Cryo-EM analyses and molecular modeling studies have shown that TTX binds to the outer vestibule of Na_v_ channels and interacts with amino acid residues around the selectivity filter through its guanidinium group and multiple hydroxyl groups [74,75,99]. The outer pore of Na_v_ channels contains the highly conserved DEKA and EEMD motifs, which together form the pore-ring architecture and contribute to toxin binding [100,101,102]. The structural environment of TTX in Na_v_PaS, an insect voltage-gated sodium channel [74], and the relative potencies of representative deoxy analogues are summarized in Figure 3. In particular, the C11 hydroxyl group has been proposed to interact with the domain IV Asp residue (D1356 in Figure 3b) in the EEMD motif [74,75,99]. This Asp residue is conserved among human Na_v_ subtypes and is also found in Na_v_ channels of diverse organisms, representing a common structural element of the TTX-binding site. TTX-resistant animals, including pufferfish, newts, and garter snakes, possess substitutions at this Asp residue or nearby residues in the outer pore region, which contribute to reduced TTX sensitivity [103,104,105,106,107]. However, because this Asp residue is also conserved in TTX-resistant Na_v_ subtypes, TTX sensitivity cannot be explained by this residue alone.

The relative potency data shown in Table 3 and Figure 3a are consistent with this binding model and indicate that the C6 and C11 substituents contribute differently to TTX toxicity. The importance of the C11 region is supported by the markedly reduced relative potency of 11-deoxyTTX (**9**), which lacks the C11 hydroxyl group [70,71,86]. In contrast, 11-oxoTTX (**3**) retains relatively high toxicity, suggesting that the presence of a polar functional group at C11 is important for maintaining favorable interactions with Na_v_ channels [78,82]. The reduced potency of 11-norTTX-6(*S*)-ol (**4**) also supports the involvement of the C11 region in potent channel-blocking activity [78]. Notably, however, this analogue still showed relatively high relative potency despite lacking the C6-equatorial-CH_2_OH group.

The C6 position also contributes to TTX toxicity. The lower relative potency of 6-deoxyTTX (**2**) compared with TTX indicates that the C6 hydroxyl group participates in TTX–Na_v_ interactions. In addition, the reduced potency of 6-*epi*TTX (**10**), in which the stereochemical arrangement of the C6-axial-OH and C6-equatorial-CH_2_OH groups is altered, indicates that the spatial arrangement around C6 is important for toxicity. Furthermore, the lower relative potency of 6,11-dideoxyTTX (**7**) compared with 11-deoxyTTX (**9**) supports the contribution of the C6-axial-OH group. The low relative potency of 6-*epi*-11-deoxyTTX (**11**) in the Neuro-2a assay can also be explained by simultaneous disruption of the interaction network around the C6 and C11 regions.

Taken together, these comparisons indicate that both the C6 and C11 substituents contribute to the high toxicity of TTX, but their contributions are not equivalent. The C11 region appears to make a major contribution to high-affinity Na_v_ binding, whereas the C6 hydroxyl group also supports toxicity through its presence and stereochemical orientation. Therefore, the relatively high toxicity of 6-deoxyTTX among deoxy-type TTX analogues is reasonable. Although loss of the C6 hydroxyl group reduces toxicity, the guanidinium-containing TTX scaffold, the C11 hydroxymethyl group, and the remaining hydroxyl groups likely maintain sufficient interactions with Na_v_ channels to support potent channel blockade. Nevertheless, toxicity data for TTX analogues remain limited, and further activity evaluations of highly purified known and new analogues using multiple assay systems are needed.

### 3.3. Occurrence of 6-deoxyTTX (***2***) in Pufferfish and Other Marine TTX-Bearing Organisms and Its Biosynthetic Implications

In the pufferfish species examined in this study, 6-deoxyTTX (**2**) was detected in a wide range of species and tissues. However, its abundance was generally low relative to that of TTX (mean 3.2%), with the exception of one sample in which the relative abundance reached 13.4%. Importantly, in this study, 6-deoxyTTX (**2**) was below the detection limit in the tissues examined from *T. rubripes*, a major edible pufferfish species. In most of the other pufferfish species examined, 6-deoxyTTX (**2**) was detected, but its abundance was generally much lower than that of TTX. Although 6-deoxyTTX (**2**) exhibited relatively high toxicity, its contribution to the overall toxicity of the pufferfish samples analyzed here is likely limited. Nevertheless, the relatively high toxicity of 6-deoxyTTX (**2**) indicates that this analogue should be included in toxin profiling. 6-DeoxyTTX (**2**) has also been detected in other marine TTX-bearing organisms. For example, 6-deoxyTTX (**2**) has been detected in the toxic flatworm *Planocerid* sp., where it accounted for only approximately 2% of the TTX content. In contrast, previous studies reported substantially higher proportions of 6-deoxyTTX (**2**) in the viscera and muscle of *Nassarius glans* (6.8–10.0% of TTX) and in the blue-ringed octopus *Hapalochlaena maculosa* (15.6% of TTX) [86]. These findings suggest that the abundance of 6-deoxyTTX (**2**) may vary considerably among species and may also be influenced by geographical location and season. Although the present study analyzed a total of 56 pufferfish tissue samples and provides an initial survey of the occurrence and distribution of 6-deoxyTTX (**2**) in pufferfish, more detailed studies using larger numbers of specimens from different locations, seasons, and tissues will be necessary to evaluate biological variation in the distribution of this analogue.

In marine organisms, 5,6,11-trideoxyTTX (**5**) is widely distributed and often occurs at relatively high concentrations. Together with the occurrence of various dideoxy-TTXs and monodeoxy-TTXs, this distribution pattern has led to the proposal of a stepwise oxidative biosynthetic pathway for TTX [67]. The detection of 6-deoxyTTX (**2**), albeit at low concentrations, across diverse pufferfish species and other toxic marine organisms provides additional support for this proposed stepwise oxidation pathway.

## 4. Materials and Methods

### 4.1. General Experimental Procedures

HR–MS spectra were obtained using a micrOTOF-QII mass spectrometer (Bruker Daltonics, Bremen, Germany), equipped with an ESI source. LC was performed on a system consisting of two LC-30AD pumps (Shimadzu, Kyoto, Japan), a SIL-30AC autosampler (Shimadzu), a CTO-20AC column oven (Shimadzu), and a CBM-20A communications bus module (Shimadzu). Mass data were acquired using otofControl 3.2,and analyzed using Bruker Compass DataAnalysis 4.1 and QuantAnalysis 2.1. Source parameters were as follows: Capillary, 4500 V; nebulizer, 1.6 bar; dry heater, 180 °C; and dry gas, 7.0 L/min (nitrogen). Representative quadrupole parameters were Collision cell RF, 120 Vpp; transfer time, 40 μs; and prepulse storage time, 5.0 μs. LC–MS-grade CH_3_CN and formic acid were purchased from FUJIFILM Wako Pure Chemical Corporation (Osaka, Japan). LC–MS-grade ammonium formate was purchased from Sigma-Aldrich (St. Louis, MO, USA). NMR spectra were recorded on an Agilent 600 MHz spectrometer (Agilent Technologies, Santa Clara, CA, USA) with a 5 mm probe at 20 °C. The ^1^H NMR spectrum was recorded using CD_3_COOD/D_2_O (4:96, *v*/*v*) as the solvent.

### 4.2. Mouse Bioassay

6-DeoxyTTX (**2**), previously isolated from the ovaries of *Takifugu pardalis*, was used in this study [86]. 6-DeoxyTTX (**2**) was confirmed by ^1^H NMR spectroscopy, and its purity was evaluated by HILIC–LC–MS (Figures S1 and S2). The administered dose was calculated based on the amount of 6-deoxyTTX (**2**), taking into account the purity of the purified sample used for the mouse bioassay (87.9%). The acute toxicity of 6-deoxyTTX was evaluated by intraperitoneal (i.p.) injection using male ddY strain mice (body weight 14.8–16.2 g) purchased from CLEA Japan, Inc. (Tokyo, Japan) according to the standard analytical methods in the food safety regulation for pufferfish toxin in Japan [93]. Because of the limited availability of purified 6-deoxyTTX (**2**), the mouse bioassay was performed with a limited number of animals. Individual mice were administered 1500 ng (*n* = 1), 1000 ng (*n* = 1), 750 ng (*n* = 2), or 500 ng (*n* = 1) of 6-deoxyTTX in 1 mL of dosing solution. The dosing solution was prepared immediately before administration by diluting the 6-deoxyTTX stock solution in dilute acetic acid (AcOH, <0.005 M AcOH) with water. Mice were continuously observed for 30 min after administration and were subsequently monitored at regular intervals until end point (24 h). The mouse unit (MU) was calculated from the body weight and time to death of each mouse according to the official tetrodotoxin toxicity conversion table specified in the Japanese standard method for pufferfish toxin analysis [93]. The mouse bioassays were approved by the Animal Ethical Committee of Tohoku University (protocol approval number 2024AgA-020).

### 4.3. Toxin Extraction and Purification

Frozen specimens of various pufferfish species stored at −20 °C were used in this study. Skin and liver tissues were collected from partially thawed specimens. Gonads were additionally collected from individuals with mature reproductive organs. Muscle tissues were also collected from *T. rubripes*. The collection site, season, sex, total length, body weight, liver weight, and gonad weight of each specimen are summarized in Table S1. In addition to individual tissue samples, four pooled ovary samples and two pooled liver samples of *T. pardalis* were analyzed. Several grams of each tissue were excised and used for toxin analysis.

Each tissue sample was finely minced with scissors and homogenized in a mortar with two volumes of 0.2 M AcOH. The homogenate was heated at 100 °C for 10 min and centrifuged at 4000× *g* for 15 min. The resulting supernatant was collected. For liver samples, an upper lipid layer formed after centrifugation and was removed prior to collection of the aqueous phase. The volume of the supernatant was adjusted to the original extraction volume with 0.2 M AcOH. An aliquot (200 μL, corresponding to 100 mg of tissue) of the extract was adjusted to pH 6 with 1 M NaOH and applied to an activated charcoal column (bed volume, 200 μL, FUJIFILM Wako Pure Chemical Corporation) packed in a Pasteur pipette. The column was washed with three volumes of water, and toxins were eluted with six volumes of 2% AcOH in 50% ethanol/H_2_O. The eluate was centrifuged at 15,000× *g* for 10 min, and a portion of the supernatant was evaporated to dryness under reduced pressure using a centrifugal evaporator. The residue was reconstituted in 0.05 M AcOH, filtered through a CosmoSpin Filter H (0.45 μm pore size, Nacalai Tesque, Kyoto, Japan), and subjected to HR-HILIC–LC–MS analysis. Extract of a planocerid flatworm collected in Guam provided by Prof. V. J. Paul and Dr. R. Ritson-Williams [5,67] was prepared using the same procedure and analyzed by HR-HILIC–LC–MS.

### 4.4. HILIC–LC–MS Analysis

HR-HILIC–LC–MS analysis was performed using a TSKgel Amide-80 column (2.0 mm × 150 mm, 5 μm; Tosoh, Tokyo, Japan) maintained at 28 °C. The mobile phase consisted of 16 mM ammonium formate buffer, CH_3_CN, and formic acid (30:70:0.02, *v*/*v*/*v*) at a flow rate of 0.2 mL/min. Extracted ion chromatograms (EICs) corresponding to the [M + H]^+^ ions of TTX analogues were analyzed at *m*/*z* 320.1088 (TTX, 4-*epi*TTX), 304.1139 (6-deoxyTTX (**2**)), 302.0983 (4,9-anhydroTTX, 4,4a-anhydroTTX). A calibration curve for TTX was prepared over the range of 5 pg (LOQ)–1000 pg (R^2^ = 0.9993). All pufferfish and flatworm extracts were analyzed in triplicate. For both TTX and 6-deoxyTTX, the LOD (3 pg) and LOQ (5 pg) were defined as signal-to-noise ratios of >5 and >10, respectively, with a coefficient of variation <20% for the standard at the LOQ. Replicate LC–MS analyses of TTX showed good reproducibility, with a mean Coefficient of Variation (CV) of 7.8% (median 5.8%, *n* = 53), and all measurements exhibited CV values below 20%. For 6-deoxyTTX (**2**), the mean CV was 11.8% (median 9.8%, *n* = 34). Although five 6-deoxyTTX measurements exhibited CV values above 20%, these corresponded to peaks near the LOQ, and the overall reproducibility was considered acceptable for trace-level quantification. Recovery experiments were performed by spiking TTX and 6-deoxyTTX (**2**) standards into skin and liver extracts of pufferfish (*Takifugu rubripes*), followed by purification using activated charcoal. The recovery rates of TTX were 88.4% and 90.5% for skin and liver extracts, respectively, whereas those of 6-deoxyTTX were 87.8% and 57.9%, respectively. Quantitative values were corrected for the recovery of the activated charcoal purification procedure. 

### 4.5. Structural Visualization of the TTX-Bound Na_v_PaS Complex

Structural visualization of the TTX-bound Na_v_PaS complex was performed using UCSF ChimeraX (version 1.11.1) [108]. The cryo-EM structure of Na_v_PaS, a voltage-gated sodium channel from the American cockroach *Periplaneta americana*, bound to TTX and gating modifier toxin Dc1a (PDB ID: 6A95) was used as a structural reference to illustrate the binding environment of TTX in the outer vestibule of Na_v_ channels [74]. Dc1a and *N*-acetylglucosamine were removed for clarity. Na_v_PaS was shown as a cartoon and transparent surface, whereas TTX and amino acid residues containing at least one atom within 4 Å of TTX were displayed as sticks. Selected polar contacts between TTX and nearby residues were indicated as cyan dashed lines in Figure 3b.

## 5. Conclusions

This study evaluated the acute toxicity and biological distribution of 6-deoxyTTX (**2**), a low-abundance TTX analogue whose toxicological significance has remained poorly understood. In the mouse bioassay, 6-deoxyTTX (**2**) was estimated to have relatively high acute toxicity compared with other reported TTX analogues, indicating its potential importance from a food safety perspective. HR-HILIC–LC–MS analysis further revealed that 6-deoxyTTX (**2**) occurs in multiple toxic pufferfish species in the genera *Takifugu*, *Arothron*, and *Lagocephalus*. In the pufferfish samples examined, 6-deoxyTTX (**2**) was generally much less abundant than TTX, suggesting that its contribution to overall toxicity is limited in these samples. Nevertheless, because of its relatively high toxicity, 6-deoxyTTX (**2**) should be regarded as a toxicologically relevant analogue. Further studies across a broader range of organisms, species, geographical regions, and seasons will be necessary for better understanding of distribution, accumulation, and biosynthetic origin of TTX-related compounds.

## Figures and Tables

**Table 1:** Results of mouse bioassays with 6-deoxyTTX (**2**).

Compound	Dose (ng)	Body Weight (g)	Lethal Time (Min:Sec), Symptoms	MU	1 MU (ng) ^a^	LD_99_ (μg/kg) ^a^
6-deoxyTTX (**2**)	1500	14.8	4:24	3.5	430	21
	1000	16.2	7:03	1.9	520	26
	750	15.2	14:00	1.0	750	38
	750	15.5	not died, severe symptoms			
	500	15.4	not died, symptoms			
Mean of lethal cases (*n* = 3)					570	28

^a^ The corresponding values for TTX (**1**) are 220 ng/MU and 10–12 µg/kg LD_99_ [45,78,93,94,95].

**Table 2:** Tissue concentrations of TTX (**1**, µg/g) and 6-deoxyTTX (**2**, ng/g) in various pufferfish species and relative abundance of 6-deoxyTTX ^a^.

Species	Tissue	TTX (µg/g)	6-deoxyTTX (ng/g)	Relative Abundance (%)
*Takifugu pardalis*	Skin	3.8	75	2.0
	Liver	6.9, 11.6, 16.1	254, 1550, 664	3.7, 13.4, 4.1
	Ovary	4.4, 6.7, 7.8, 18.3	LOD ^d^, LOQ ^f^, 446, 366	-, -, 5.7, 2.0
*T*. *snyderi*	Skin	5.0	42	0.84
	Liver	4.6	61	1.3
	Testis	2.5	LOD ^d^	-
*T*. *niphobles*	Skin	1.7, 2.8, 3.8	LOD ^e^, 43, 87	-, 1.5, 2.3
	Liver	1.5, 2.1, 3.4	LOQ ^f^, 89, 89	-, 4.2, 2.7
	Ovary	4.2, 6.5	180, 264	4.2, 4.1
	Testis	4.4	135	3.1
*T*. *exascurus*	Skin	2.2, 3.6, 4.3	38, 99, 77	1.7, 2.8, 1.8
	Liver	2.0, 2.2, 2.5	LOQ ^f^, 123, 98	-, 5.6, 3.9
	Ovary	5.2	181	3.5
*T*. *flavipterus*	Skin	6.4, 13	90, 378	1.4, 2.9
	Liver	3.2, 4.4	136, 81	4.2, 1.8
	Ovary	14	712	5.1
*T*. *rubripes*	Skin	LOQ ^c^, 0.093	LOD ^e^	-, -
	Liver	LOD ^b^, 0.13	LOD ^d^	-, -
	Muscle	LOD ^b^, LOQ ^c^	LOD ^e^	-, -
*T*. *vermicularis*	Skin	0.71, 0.84, 1.8	LOD ^e^	-, -, -
	Liver	0.30, 0.65, 0.92	LOD ^d^	-, -, -
*Arothron hispidus*	Skin	7.6, 203	74, 466	0.97, 0.23
	Liver	0.15, 1.7	LOD ^d^	-, -
	Ovary	4.3	117	2.7
*A*. *manilensis*	Skin	1.2, 78	LOD ^e^, 1802	-, 2.3
	Liver	1.4, 4.2	LOD ^d^, 66	-, 1.6
	Ovary	6.7	69	1.0
*Lagocephalus sceleratus*	Skin	1.8	114	6.3
	Liver	1.1	LOQ ^f^	-

^a^ When multiple values are shown in a row, the values correspond to individual samples and are listed in the same order across the TTX, 6-deoxyTTX, and relative abundance columns. ^b–f^ The LOD for TTX was 20 ng/g in both liver/gonad and skin/muscle tissues (b). The limit of quantification (LOQ) for TTX was 34 ng/g in skin/muscle tissues (c). The LODs for 6-deoxyTTX were 31 ng/g in liver/gonadal tissues (d) and 21 ng/g in skin/muscle tissues (e), and the LOQ for 6-deoxyTTX was 52 ng/g in liver/gonadal tissues (f).

**Table 3:** Relative toxicities of TTX analogues evaluated by mouse bioassay and Neuro-2a assay ^a^.

	Mouse Bioassay (i.p.)				Neuro-2a Assay		
Compound	LD_50_ (μg/kg)	LD_100/99_ (μg/kg)	Relative Potency	Ref.	EC_50_ (nM)	Relative Potency	Ref.
TTX (**1**)	10	10–12	1	[45,78,95]	1.9–8.4	1	[73,77,78,82,86,92]
6-deoxyTTX (**2**)		28	0.36	This study	6.5	0.32	[86]
11-deoxyTTX (**9**)	71		0.14	[87]	130, 270	0.02, 0.02	[86,92]
6,11-dideoxyTTX (**7**)	420		0.02	[85]	400	0.05	[86]
5,6,11-trideoxyTTX (**5**)			0.01 ^b^	[83]			
11-oxoTTX (**3**)		16, 24	0.63, 0.42	[78,80]	2.8, 2.9	3.0, 1.6	[78,82]
11-norTTX-6(*S*)-ol (**4**)		20, 54	0.50, 0.19	[78,98]	178	0.05	[78]
6-*epi*TTX (**10**)	60		0.17	[87]	33	0.06	[73]
6-*epi*-11-deoxyTTX (**11**)					187	0.03	[77]

^a^ The relative potencies were calculated by comparison with TTX using the corresponding toxicity parameters reported for each assay. Blank cells indicate that no corresponding value was available. ^b^ Calculated based on the minimum lethal dose (750 μg/kg).

## Data Availability

Data are available only in this article.

## Supplementary Materials

The following supporting information can be downloaded at https://www.mdpi.com/article/10.3390/md24070250/s1, Table S1: Collection site, season, sex, body size and tissue weights of the pufferfish specimens used in this study; Figure S1: Extracted ion chromatograms (EICs) of the purified 6-deoxyTTX (**2**) used in the mouse bioassay, acquired in positive-ion mode. TTX and 4,9-anhydroTTX were not detected above the limit of detection (LOD); Figure S2. ^1^H NMR spectrum of purified 6-deoxyTTX (**2**) used in the mouse bioassay. Signals assigned to the hemilactal form are annotated, and signals attributable to the 10,7-lactone form were also observed [86]. Residual solvent was removed by repeated evaporation prior to the assay.

## Abbreviations

The following abbreviations are used in this manuscript:

TTX	tetrodotoxin
EFSA	European Food Safety Authority
NMR	nuclear magnetic resonance
HILIC	hydrophilic interaction liquid chromatography
LC–MS	liquid chromatography–mass spectrometry
HR	high resolution
LOD	limit of detection
LOQ	limit of quantification
EIC	extracted ion chromatogram
Na_v_	voltage-gated sodium channels
Cryo-EM	cryo-electron microscopy
i.p.	intraperitoneal
MU	mouse unit
LD_99_	99% lethal dose
LD_50_	median lethal dose
EC_50_	half-maximal effective concentration
IC_50_	half-maximal inhibitory concentration
AcOH	acetic acid
