# Supplementary material for: Toxicity Assessment of 6-Deoxytetrodotoxin by Mouse Bioassay and Its Distribution in Pufferfish
Source: Mar Drugs. 2026 Jul 18;24(7):250. doi: 10.3390/md24070250 (PMC13412326; doi:10.3390/md24070250)
Supplement: Supplementary file 1 [file marinedrugs-24-00250-s001.zip › marinedrugs-4416142-supplementary.pdf]

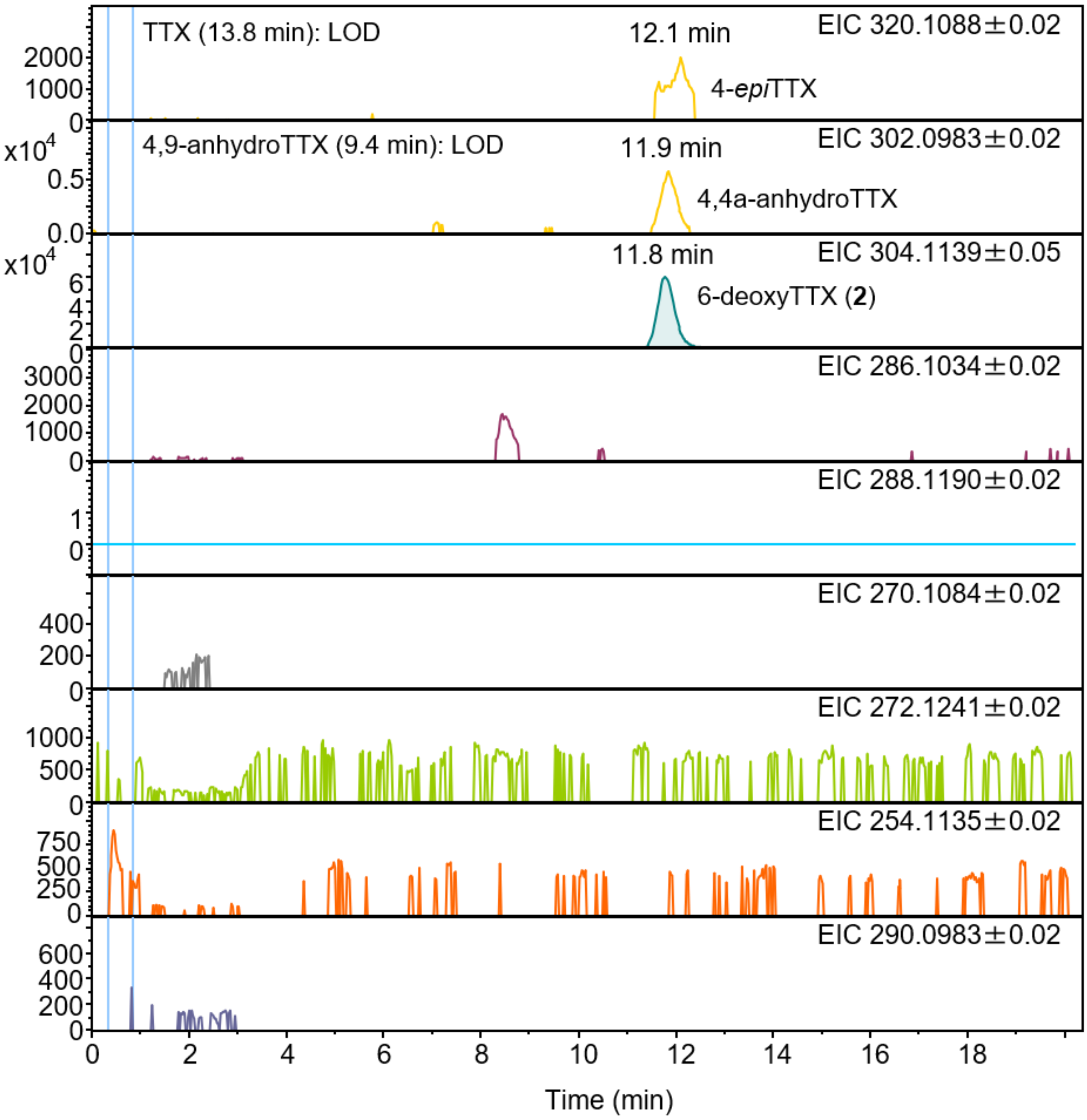

Figure S1. Extracted ion chromatograms (EICs) of the purified 6-deoxyTTX (**2**) used in the mouse bioassay, acquired in positive-ion mode. TTX and 4,9-anhydroTTX were not detected above the limit of detection (LOD).

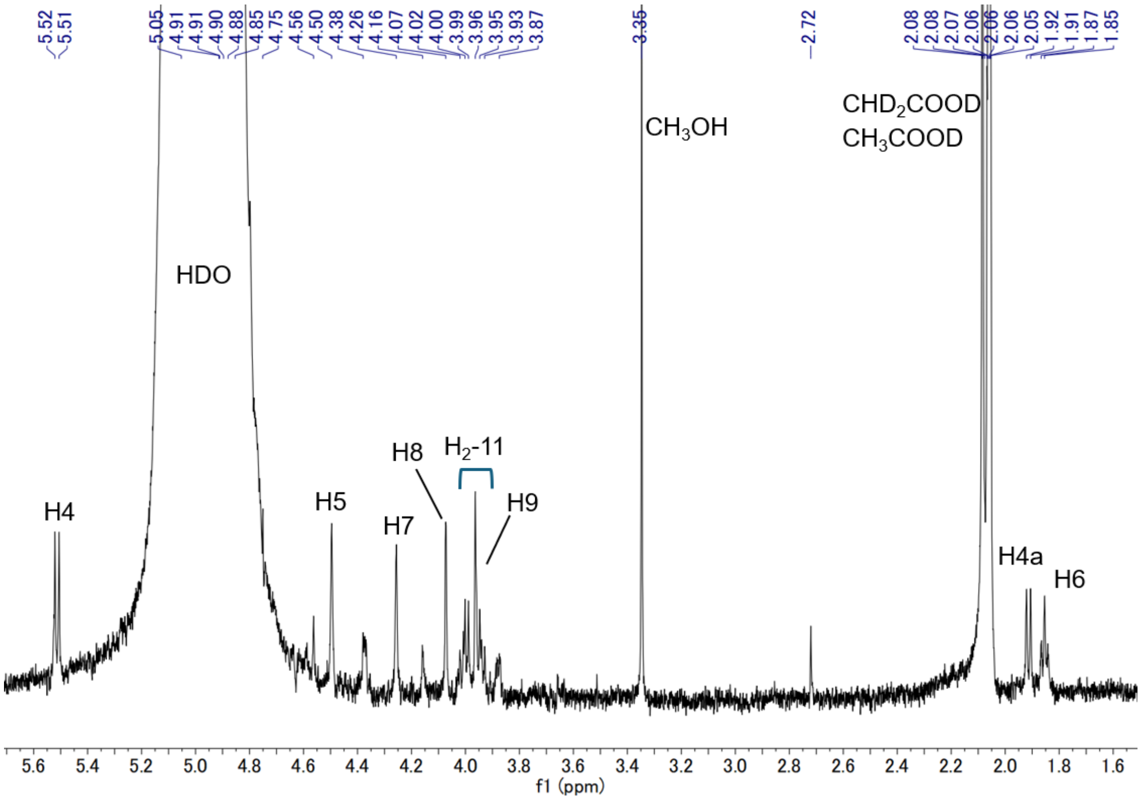

Figure S2. ^{1}H NMR spectrum of purified 6-deoxyTTX (**2**) used in the mouse bioassay. Signals assigned to the hemilactal form are annotated, and signals attributable to the 10,7-lactone form were also observed [1]. Residual solvent was removed by repeated evaporation prior to the assay.

1. Kudo, Y.; Finn, J.; Fukushima, K.; Sakugawa, S.; Cho, Y.; Konoki, K.; Yotsu-Yamashita, M. Isolation of 6-Deoxytetrodotoxin from the Pufferfish, *Takifugu pardalis*, and a Comparison of the Effects of the C-6 and C-11 Hydroxy Groups of Tetrodotoxin on Its Activity. *J. Nat. Prod.* **2014**, *77*, 1000–1004.

Table S1. Collection site, season, sex, body size and tissue weights of the pufferfish specimens used in this study.

	Collection site (Japan)	Collection month	Sex	Total length (cm)	Body weight (g)	Liver (g)	Ovary (g)	Testis (g)
Takifugu pardalis	Mie Prefecture	May	Not determined	20.0	144.6	6.7		
Takifugu snyderi	Mie Prefecture	May	Male	23.5	262.1	3.8		37.2
Takifugu niphobles	Mie Prefecture	May	Female	15.0	123.7	4.6	34.4	
	Mie Prefecture	May	Male	16.5	120.0	4.0		22.4
	Mie Prefecture	May	Female	17.5	99.2	5.5	7.5	
Takifugu exascurus	Mie Prefecture	May	Female	19.0	152.2	8.8	4.8	
	Mie Prefecture	May	Not determined	20.5	129.5	5.9		
	Mie Prefecture	May	Not determined	15.0	57.0	2.8		
Takifugu flavipterus	Mie Prefecture	May	Female	22.5	176.2	6.4	28.8	
	Mie Prefecture	May	Not determined	16.5	77.1	1.9		
Takifugu rubripes (wild-caught)	Miyagi Prefecture	October	Not determined	30.0	821.8	44.3		
	Miyagi Prefecture	October	Not determined	45.0	1613.9	126.7		
Takifugu vermicularis	Miyagi Prefecture	May	Not determined	26.0	299.0	18.9		
	Miyagi Prefecture	May	Not determined	27.0	300.2	18.8		
	Miyagi Prefecture	May	Not determined	25.5	311.5	23.3		
Arothron hispidus	Okinawa Prefecture	June	Not determined	29.0	487.4	72.8		
	Okinawa Prefecture	June	Female	47.0	2550.0	87.0	513.0	
Arothron manilensis	Okinawa Prefecture	June	Female	17.5	294.6	10.9	181.4	
	Okinawa Prefecture	April	Presumed male	22.0	273.0	20.7		
Lagocephalus sceleratus	Izu city, Shizuoka Prefecture	May	Presumed male	33.0	430.4	16.8		
